# Hydrocarbon bio-jet fuel from bioconversion of poplar biomass: techno-economic assessment

**DOI:** 10.1186/s13068-016-0545-7

**Published:** 2016-06-23

**Authors:** Jordan T. Crawford, Chin Wei Shan, Erik Budsberg, Hannah Morgan, Renata Bura, Rick Gustafson

**Affiliations:** 1 University of Washington, Seattle, WA USA; 2 School of Environmental and Forest Sciences, University of Washington, Box 352100, Seattle, WA 98195-2100 USA

**Keywords:** Techno-economics, Hydrocarbon biofuel, Biorefinery, Economics, Acetogen, Lignin gasification, Hydrogen

## Abstract

**Background:**

Infrastructure compatible hydrocarbon biofuel proposed to qualify as renewable transportation fuel under the U.S. Energy Independence and Security Act of 2007 and Renewable Fuel Standard (RFS2) is evaluated. The process uses a hybrid poplar feedstock, which undergoes dilute acid pretreatment and enzymatic hydrolysis. Sugars are fermented to acetic acid, which undergoes conversion to ethyl acetate, ethanol, ethylene, and finally a saturated hydrocarbon end product. An unfermentable lignin stream may be burned for steam and electricity production, or gasified to produce hydrogen. During biofuel production, hydrogen gas is required and may be obtained by various methods including lignin gasification.

**Results:**

Both technical and economic aspects of the biorefinery are analyzed, with different hydrogen sources considered including steam reforming of natural gas and gasification of lignin. Cash operating costs for jet fuel production are estimated to range from 0.67 to 0.86 USD L^−1^ depending on facility capacity. Minimum fuel selling prices with a 15 % discount rate are estimated to range from 1.14 to 1.79 USD L^−1^. Capacities of 76, 190, and 380 million liters of jet fuel per year are investigated. Capital investments range from 356 to 1026 million USD.

**Conclusions:**

A unique biorefinery is explored to produce a hydrocarbon biofuel with a high yield from bone dry wood of 330 L t^−1^. This yield is achieved chiefly due to the use of acetogenic bacteria that do not produce carbon dioxide as a co-product during fermentation. Capital investment is significant in the biorefinery in part because hydrogen is required to produce a fully de-oxygenated fuel. Minimum selling price to achieve reasonable returns on investment is sensitive to capital financing options because of high capital costs. Various strategies, such as producing alternative, intermediate products, are investigated with the intent to reduce risk in building the proposed facility. It appears that producing and selling these intermediates may be more profitable than converting all the biomass into aviation fuel. With variability in historical petroleum prices and environmental subsidies, a high internal rate of return would be required to attract investors.

**Electronic supplementary material:**

The online version of this article (doi:10.1186/s13068-016-0545-7) contains supplementary material, which is available to authorized users.

## Background

In 2012, ethanol comprised 94 % of U.S. biofuel production, most of which was derived from corn starch [1]. The same year, a study on the U.S. gasoline market shows it is already saturated with 10 % ethanol blends, and that widespread use of blends higher than 10 % such as E15 will require existing infrastructure be upgraded or replaced [2]. It is apparent that producing additional ethanol for liquid transportation fuel may be undesirable compared to infrastructure compatible biofuels such as hydrocarbons, which will avoid some of the problems associated with ethanol production in the U.S. If produced in an economically competitive manner, hydrocarbon biofuels have great potential to displace petroleum derived transportation fuels.

Techno-economic modeling is a useful tool to study the workings of capital projects. In short, techno-economics investigate the technical performance and economic potential of proposed scenarios. Analyses have been performed for various clean energy-related projects, such as biodiesel production [3], hydrogen production [4], hydrogen sourced power generation [5], carbon dioxide capture from coal fired power plants [6], and fast pyrolysis of biomass for fuel and energy [7, 8]. The techno-economic field combines engineering and economics—a multi-disciplinary approach necessary for any technology to transition from research to industry.

Specifically when looking at transportation fuels, lignocellulosic ethanol production is the topic of many techno-economic analyses [9]. As a more stringent subset of transportation fuels, aviation biofuels have perhaps been studied less frequently. However, various pathways such as gasification and Fischer–Tropsch, hydrothermal liquefaction, pyrolysis, direct sugars to hydrocarbons, hydroprocessed esters and fatty acids, and alcohol-to-jet have been explored [10–13]. Feedstock choice affects techno-economic outcomes, as softwoods, hardwoods, and agricultural residues have different results in ethanol production [14]. Pretreatment is the subject of intense research with many competing technologies, such as dilute sulfuric acid, sulfur dioxide catalyzed steam explosion, controlled pH, ammonia fiber expansion, or lime pretreatment [15]. Regarding enzymatic hydrolysis, simultaneous saccharification and fermentation, or separate hydrolysis and fermentation can be used [16]. After sugars are liberated from lignocellulosic material, the fermenting organism affects yields. A range of bacteria, yeasts, and fungi have been investigated, some naturally occurring and some recombinant, for ethanol production from lignocellulosic hydrolysate [17]. The complex biofuels factory uses mechanical, biological, and chemical platforms in an effort to maximize yield from feedstock and minimize cost.

The goal of the present study is to first create a representative model of a unique biofuels production method that uses acetic acid fermentation. The novelty and main advantage of this method is the 50 % theoretical increase in maintaining carbon in the process stream with acetic acid fermentation compared to ethanol fermentation, based on stoichiometric conversion. The biorefinery, so-called due to the multiple intermediate chemicals in the process stream, is envisioned as a near-term technology. As will later be described in detail, the process ferments acetic acid from hydrolyzed biomass sugars, and through chemical conversion produces an ethanol intermediate and hydrocarbon biofuel end product. This techno-economic analysis agglomerates process information from the public domain, which is set into process simulation software Aspen Plus. Quantitative results are generated, including mass, energy, and work flows. This information is available for multiple uses including environmental analyses, such as life cycle assessments, and economic analysis. The goals of economic analysis are to estimate both capital and operating expenses of the biorefinery and to assess the minimum product selling price to achieve a target rate of return. Economies of scale are investigated using the techno-economic method to generate data for multiple capacity biorefineries. In this paper, we analyze three different biorefineries with capacities of 76, 190, and 380 ML y^−1^.

## Results and discussion

The results of the Aspen simulations of the various process scenarios detailed in “Methods” were used to assess technical feasibility and economic viability. The following details the results of the technical and economic analyses.

### Process analysis

Analysis of process simulation gives insights into technical feasibility of different processes. Hydrogen, steam, and electricity play a large interconnected role within the biorefinery, and are analyzed here.

Natural gas reforming and lignin gasification processes to produce hydrogen were simulated. Natural gas reforming is less complex, and much more industrially proven than lignin gasification. A noteworthy amount of hydrogen is consumed in jet fuel production for the bioconversion process envisioned in this research; H_2_ is consumed at a rate of approximately 0.12 kg L^−1^ of jet fuel. The complexity of lignin gasification makes this a questionable process for hydrogen production unless there are compelling economic or environmental reasons to adopt this technology.

Steam and heat play a critical role in this biorefinery, as it would in most configurations. The following tables show steam consumption and related electricity production, based on a 76 ML y^−1^ facility. Table 1 summarizes consumption in major steam consumer unit operations. An additional file shows the Aspen operating temperatures and pressures of the major unit operations (see Additional file 1). Pretreatment is the largest consumer and, due to the steam being injected directly into the biomass process stream, no condensate is returned. Large volumes of high pressure steam are required for the ethanol to ethylene reactor, but this condensate may be returned to the boiler reducing the overall water usage.

**Table 1:** Major biorefinery unit operation steam users per liter of jet fuel produced

Process	Steam usage (kg)	Steam temperature (°C), pressure (kPa)
Pretreatment	2.1	280, 1310
Pressure swing distillation column (1)	0.4	280, 1310
Pressure swing distillation column (2)	1.1	280, 1310
Ethanol to ethylene	1.3	580, 450

Table 2 shows electricity consumption by process area. The last two items cover hydrogen production and differ substantially due to syngas compression. In natural gas reforming, the gas feed is already pressurized, whereas for lignin gasification the lignin feed is at near atmospheric pressure and energy must be utilized to pressurize the system. The results of this systematic disparity come to light in Table 3, where net electricity production is shown. In the case where hydrogen is derived from natural gas reforming, excess electricity is produced and may be sold. When hydrogen is derived via lignin gasification, a deficit of electricity exists and so must be purchased. While the difference is relatively small, it will impact both economics and environmental assessments.

**Table 2:** Biorefinery electricity consumption by area per liter of jet fuel of produced

Process	kWh
Feedstock handling	0.03
Pretreatment	0.20
Hydrolysis and fermentation	0.10
Ethylene compressor	0.11
Wastewater treatment	0.11
Utilities	0.11
Boiler area	0.05
Natural gas reforming	0.06
Lignin gasification	0.38

Two scenarios (natural gas reforming and lignin gasification) are shown for hydrogen production

**Table 3:** Net biorefinery electricity per liter of jet fuel produced

	Natural gas reforming (kWh)	Lignin gasification (kWh)
Consumed	0.93	1.07
Generated	0.98	1.0
Net	0.05	−0.07

Shown are two scenarios of hydrogen production

Net electricity production in the envisioned biorefineries is low compared to similar studies that produce ethanol [18] instead of a hydrocarbon. This is due to the extra processing steps involved in the acetogen production pathway to produce ethanol, additional processing steps to make a hydrocarbon fuel instead of only ethanol, and the necessary hydrogen production. The low electricity production reduces the income and profitability of the biorefinery and may affect life cycle assessment results due to excess electricity not being able to displace fossil fuel-produced electricity [19].

### Expense estimates

Capital costs are estimated as described in “Methods.” Table 4 shows costs of a 380 ML y^−1^ facility broken down into major process areas. A comparison is made for the two hydrogen production scenarios of natural gas reforming and lignin gasification. Proportionally, the front end of the process, converting biomass to ethanol, is the most expensive and riskiest part of the process. This is due in part to the additional processing steps necessary to get to an alcohol when fermenting acetic acid instead of ethanol. Additional capital expense is derived from the extra processing steps necessary to produce a hydrocarbon end product. An ethanol producing facility may have a capital cost of 1.83 USD per annual liter with a capacity of 230 ML y^−1^ [18], and an alcohol-to-jet facility using four carbon alcohols may have costs of 4.03 USD per annual liter with a capacity of 70 ML y^−1^ [10]. The present study estimates costs of 4.70, 3.14 and 2.37 USD per annual liter of jet fuel for capacities of 76, 190, and 380 ML y^−1^, respectively, using natural gas reforming. These numbers are higher due to the previously mentioned additional complexities of fermenting acetic acid, and taking the product to jet fuel when compared to ethanol.

**Table 4:** Capital costs for 380 million liter per year jet fuel facility

Fixed capital (million USD)	Natural gas reforming	Lignin gasification
Feedstock handling	127	127
Pretreatment	130	130
Hydrolysis and fermentation	151	151
Reactive distillation and alcohol separation	124	124
Alcohol to hydrocarbon	82	82
Hydrogen production (reforming/gasification)	73	197
Steam plant	78	78
Utilities	9	9
Waste water treatment	129	129
Total	$902	$1026

The costs are broken down into major process areas for comparison purposes

**Fig. 1:**
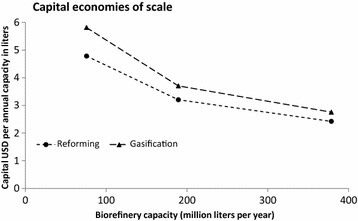
Economies of scale of capital expense for three different capacity biorefineries

The factored estimation method is applied to calculate the capital cost of three different capacity biorefineries. Economies of scale can be seen in the range of capacities investigated for facilities producing 76–380 ML y^−1^ of biofuel. This is shown in Fig. 1 for the two different cases of hydrogen production, where the capital dollars per biorefinery capacity is plotted against the capacity. In Table 5, the absolute capital cost estimates are shown.

**Table 5:** Fixed capital costs of biorefineries of various capacity

Capacity (ML y^−1^)	76	190	380
Reforming capital (million USD)	356	596	902
Gasification capital (million USD)	433	690	1026

Table 6 shows an operating expense breakdown in USD for a facility producing jet fuel at a rate of 380 ML y^−1^. Feedstock is assumed to be bought at the facility gate, chipped, at 77 USD t^−1^ (dry weight), and natural gas is priced at 0.0033 USD MJ^−1^ on a higher heating value basis. Electricity is sold at 0.06 USD kWh^−1^. All assumptions for the discounted cash flow analysis are discussed in “Methods,” where 7 and 15 % discount rates are used. In the case of natural gas reforming for hydrogen production, a portion of the heat required by the biorefinery cannot be provided by the lignin stream alone. So, an additional heating medium is required. Natural gas is chosen as one option, and a biomass-based hog fuel is chosen as an alternative. Assuming a purchase price of $55 t^−1^ and energy of 11 MJ kg^−1^, purchasing hog fuel for a small portion of the biorefinery heat duty results in essentially the same operating costs as natural gas. One major benefit of using hog fuel is the reduction in overall fossil fuel use to produce the biofuel.

**Table 6:** Cash operating cost and minimum fuel selling price for 380 million liter per year jet fuel facility

Operating cost	USD per liter	
	Reforming	Gasification
Feedstock	0.23	0.23
Cellulase enzymes	0.13	0.13
Fermentation nutrients	0.01	0.01
Other raw materials	0.09	0.09
Waste disposal	0.01	0.01
Electricity	−0.02	0.004
Reforming/Gasification O&M	0.01	0.01
Natural gas (and hog fuel)	0.07	0.07
Fixed manufacturing costs	0.14	0.15
Total cash cost	0.67	0.70
Minimum selling price with 7 % discount	0.93	0.99
Minimum selling price with 15 % discount	1.13	1.22

Operating costs are broken down into major groupings

**Fig. 2:**
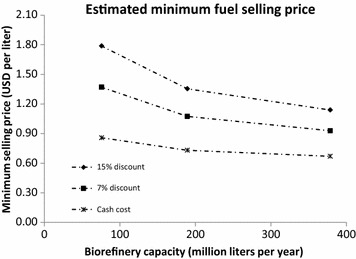
Operating expense estimates. The cash cost is shown in addition to minimum selling prices using 7 and 15 % discount rates. The costs shown use natural gas reforming as a hydrogen source

Minimum selling prices are plotted in Fig. 2, where economies of scale become apparent as plant capacity increases. As the discount rate increases, economies of scale also become more pronounced. Cash cost is defined here as the production cost to make 1 L of hydrocarbon fuel, not including any discount, tax, capital depreciation, or other factors. It includes only the operating costs, both fixed and variable, and includes any credit for electricity sold. For comparison, a 2015 techno-economic study estimated minimum fuel selling prices near 1.28 USD L^−1^ using hydrothermal liquefaction, 1.46 USD L^−1^ using pyrolysis, and 2.28 USD L^−1^ using alcohol-to-jet pathways for facilities with capacities around 150 ML y^−1^ and a discount rate of 10 % [12], assuming a fuel density of 0.8 kg L^−1^, and a currency conversion of 1.14 USD per euro. Additionally, another alcohol-to-jet study in 2015 estimated minimum fuel selling prices near 1.46 USD L^−1^ for a facility with a capacity of around 70 ML y^−1^ and a discount rate of 10 % [10], again using a currency conversion of 1.14 USD per euro. The global average price paid at the refinery for aviation jet fuel for 2013—when this analysis was first completed—was 0.78 USD L^−1^, but in 2015 was under 0.40 USD L^−1^ [20].

### Sensitivity

The previously shown results are a case study, and many variables affect economics. A brief sensitivity analysis is conducted to look at changes in minimum fuel selling price relative to feedstock cost, enzyme cost, and equity financing.

**Fig. 3:**
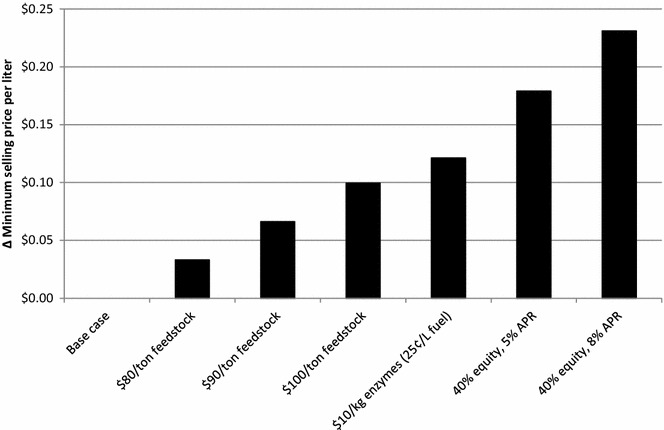
Change in minimum jet fuel selling price for feedstock, enzyme, and equity variations. The additional cost shown in the figure compares to a base case described in this paper, and is based on a discounted cash flow rate of return analysis with a 15 % nominal discount rate, feedstock price paid of 77 USD t^−1^, enzyme cost of 5.22 USD kg^−1^ (0.13 USD L^−1^ fuel), and equity share of 100 %

Figure 3 compares the minimum selling prices with a 15 % discount rate to feedstock cost, enzyme cost, and equity financing variables. The base case, shown in Table 6, assumes purchase delivered cost of feedstock at 77 USD t^−1^, enzymes at 5.22 USD kg^−1^ (0.13 USD L^−1^ fuel), and a 100 % equity share. We show what the change in minimum selling price would be per liter of jet fuel if feedstock were purchased at 88, 99 USD t^−1^, or 110 USD t^−1^. Additionally, we investigate the price increase if hydrolysis enzymes are purchased for 10 USD kg^−1^. Finally, we look at two scenarios where project equity is 40 %, with annual percentage rates (APR) of charge at 5 and 8 % for borrowed money. It can be seen that the impacts of having to borrow capital for construction of the biorefinery are greater than potential increases in critical operating costs. The relatively large increase in minimum selling price when a significant portion of the capital cost must be borrowed shows how the high capital cost impacts the overall process economics. It is similarly noted in other alcohol-to-jet studies that capital cost has a considerable effect on jet fuel production cost [10]. In summary, given the relatively prominent variations in the minimum selling price, a production biorefinery must be vigilant in controlling operating and capital expenses.

### Alternative products

While this analysis focuses on jet fuel production, it is worth noting that several chemical intermediates are marketable products themselves. Acetic acid, ethyl acetate, ethanol, and ethylene are all intermediates to polymer jet fuel production. Separation and purification to a chemical commodity purity are not explored here, although the biorefinery as envisioned already has relatively pure streams of ethyl acetate, ethanol, and ethylene. We envision that the recovery of these streams would be relatively straightforward from an engineering perspective. Producing and selling more than one product reduces risk in the biorefinery via product diversification.

Table 7 explores theoretical revenues of four different biorefineries built with the capacity to process 1.13 Mt y^−1^ of bone dry biomass. Gross revenues are shown for converting 1.13 Mt y^−1^ into jet fuel, ethanol, ethylene, or ethyl acetate. Given the estimated selling prices [20, 21] revenue could increase compared to producing only jet fuel. Capital cost variations are not explored here, although it should be recognized that both positive and negative implications exist via fewer processing steps when not going all the way to jet fuel, and extra equipment necessary for alternative product purification. It can be seen in Table 7 that selling some chemical intermediates could be considerably more profitable than converting all the biomass into a final jet fuel product.

**Table 7:** Alternative products

Product	Annual capacity	Estimated selling price	Gross revenue (million USD)
Jet fuel	380 ML	0.80USD L^−1^ [20, 21]	300
Ethanol	600 ML	0.55 USD L^−1^ [20, 21]	330
Ethylene	285,000 t	1320 USD t^−1^ [20, 21]	375
Ethyl acetate	475,000 t	1320 USD t^−1^ [20, 21]	625

A facility with the capacity to process 1.13 Mt y^−1^ of bone dry biomass could produce any one of the products. Shown here are four different setups a single biorefinery could have, but do not take into consideration any capital cost implications of biorefinery modifications. The table shows gross revenue given the referenced selling price

## Conclusions

A unique biorefinery is explored to produce a hydrocarbon biofuel as part of the desire for infrastructure compatible cellulosic biofuels. A high hydrocarbon biofuel yield of 330 L t^−1^ feedstock is achieved in large part due to the use of acetogenic bacteria that do not produce carbon dioxide as a co-product during fermentation. Compared to an ethanologen process, the present biorefinery has more unit operations and goes through more intermediate products to produce ethanol. From ethanol, a polymer fuel is produced further increasing the complexity of the biorefinery to make a more valuable and infrastructure compatible end product.

A challenge in the process is sourcing hydrogen required for the biorefinery. With the bulk of the current hydrogen in the world being produced from natural gas, a fossil fuel, the environmental impact and expense of the hydrogen must be considered when looking at the overall biofuel produced. While the additional operating expenses of hydrogen production are relatively small (about 15 % of total cash operating expenses), it does add significant capital expense as well as a degree of complexity to the biorefinery. Lignin gasification for hydrogen is more complex and capital intensive, and is an unproven technology with higher risk. Additionally, it leaves the biorefinery with a lack of heating source for process heat and steam.

The substantial capital cost leads to a high minimum selling price sensitivity when considering financing options. Strong economies of scale are shown. We also show that process economics have the potential to improve with alternative products. From an operating expense standpoint, we show that the cash production cost is marketable at conditions prior to 2015. To achieve a reasonable return, however, the minimum selling price is near the highest level that has been historically observed. We estimate the cash operating cost to be 0.67 USD L^−1^ at the cheapest configuration, a 380 ML y^−1^ facility that uses natural gas reforming for hydrogen production. A discount rate of 7 % makes the minimum selling price 0.93 USD L^−1^, which is higher than the 2013 average price paid at the refinery of 0.78 USD L^−1^ and about twice the 2015 price of about 0.40 USD L^−1^ [20]. A discount rate of 15 % increases the minimum selling price even further to 1.14 USD L^−1^. Although we did not consider any carbon tax or government incentives, these could increase profitability of jet fuel production in the short term. To this end, an in-depth analysis of the life cycle assessment of the biofuel production process detailed in this paper is performed by Budsberg et al. in an accompanying paper [22]. As briefly explored here, alternatives such as displacing some natural gas use with hog fuel are considered with respect to global warming potential.

Considering the significant capital investment required in building these facilities, the previous 20 years’ volatility in petroleum prices, and the political ebb and flow that can be associated with subsidies or a carbon tax, building a biorefinery as described would incur a large amount of risk and necessitate a high internal rate of return to attract private investors. However, there are ways to reduce risk such as selling more than one product at a time and netting a premium for “green” fuels and chemicals. Future work may investigate in further detail where to build these biorefineries and what, if any, incentives are required.

## Methods

### Hydrocarbon biofuel production

The following section describes a biorefinery that we envision could become a commercial technology. The overall process is described with one process train in mind for the sake of analysis, even though certain unit operations have interchangeable technologies. For example, one could use any number of pretreatment options. While this biorefinery is hypothetical, it represents a possible configuration for future commercial processes.

### Hydrocarbon biofuel production: feedstock

Short rotation woody crops, such as hybrid poplar, present an attractive option for diversifying and expanding biomass for biofuel production. Already used for various end-uses such as fuel wood, lumber, and paper, hybrid poplar is a well-established crop with good characteristics for biofuel use. In general, it requires little fertilizer input, can be cultivated on marginal lands, has the ability to re-sprout after multiple harvests, and has high biomass production [19, 23–25]. The lignocellulosic material in the wood can also be fractionated without extensive pretreatment [26] and hardwoods do not exhibit the recalcitrance reported in softwoods [27].

Hybrid poplar is chosen as a feedstock in part due to its good growing characteristics and ability to grow at a fast rate and in diverse geographical areas [28]. Yet another advantage of hybrid poplar is its ability to be harvested at any time of the year. This means that the biomass is stored in the field, on the stump, so that it can be harvested when needed by the biorefinery. Just in time harvesting reduces both the need for storage infrastructure at the biorefinery, and biomass degradation that occurs during long-term storage. The composition assumed for biomass, which falls within ranges given in literature [28], entering the biorefinery is shown in Table 8. Other minor components of the biomass (by dry mass) include extractives (4.5 %), acetate (2.9 %), and ash (1.9 %). It is assumed that the biomass enters the biorefinery as 50 % water by mass.

**Table 8:** Cellulose, hemicellulose, and lignin content in poplar feedstock, comprising 91 wt% of the biomass

Cellulose (% dry wt.)	42.0
Hemicellulose (% dry wt.)	22.9
Lignin (% dry wt.)	25.8

Other minor components are given in text

### Hydrocarbon biofuel production: feedstock conversion to acetic acid

**Fig. 4:**
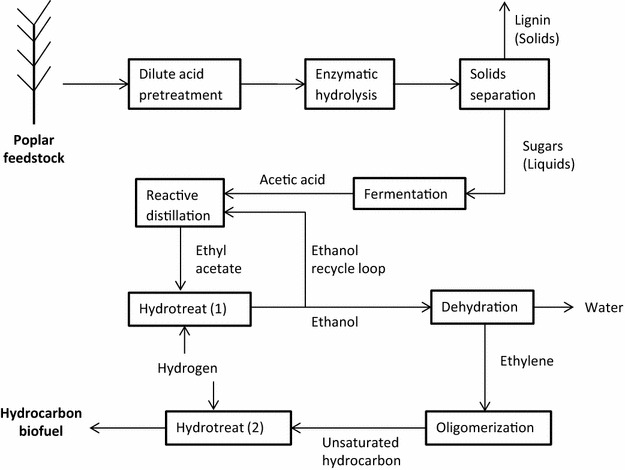
Generalized flow diagram of biorefinery. This biorefinery ferments sugars to acetic acid instead of ethanol. Ethyl acetate, ethanol, and ethylene are major intermediate products towards a hydrocarbon, polymer jet fuel product. Yields are shown in Table 9

Starting with chipped hybrid poplar, the first section of the process ends with a dilute fermentation product stream created by fermenting hydrolyzed cellulosic sugars. These process steps are outlined in Fig. 4. Most of the steps are commonly modeled [18, 29], with the exception that the fermentation product is usually ethanol instead of acetic acid.

The first two major unit operations, pretreatment and hydrolysis, convert raw cellulosic biomass into fermentable sugars. Dilute acid pretreatment is used, and the stream is then cooled and pH adjusted for enzymatic hydrolysis where cellulose is converted into fermentable sugars. Enzymes are purchased externally for this analysis rather than being produced within the biorefinery. Next, most of the insoluble solids, comprised chiefly of lignin, are separated from the liquid stream.

As opposed to other cellulosic ethanol processes, an acetogenic bacterium is employed to ferment hydrolyzate rather than an ethanologen. To maintain an acceptable pH for fermentation, the acetate product is neutralized by the addition of calcium carbonate. A solution of about 5 wt% calcium acetate leaves in the fermentation broth. A final solid–liquid separation takes place to separate out bulk biomass, which may be performed with a cross-flow filtration system.

From a carbon perspective, the process is very efficient and maintains a high percentage of feedstock carbon in the end product. *Moorella thermoacetica*, previously known as *Clostridium thermoaceticum*, ferments both five and six carbon sugars with acetate as the only product [30]. This compares to fermentation with *Saccharomyces cerevisiae*, where two-thirds of the carbon in a molecule of glucose is kept in the ethanol product, and the remaining third is fermented to the greenhouse gas carbon dioxide. With the exception of cell reproduction, the acetogenic bacterium theoretically has 100 % efficiency in maintaining the carbon in the process stream. Equation 1 shows the overall chemical equation to produce ethanol from sugar using an acetogen, and includes methane as a source of hydrogen (hydrogen consumption is discussed in later sections.) On a molar sugar basis, the two fermentation pathways can be compared, showing that theoretical carbon dioxide emissions are 25 % lower, while ethanol yields are 50 % higher using an acetogen instead of an ethanologen [Eq. 2].

1 $${\text{C}}_{ 6} {\text{H}}_{ 12} {\text{O}}_{6} + 1.5 {\text{CH}}_{4} \to 3 {\text{C}}_{ 2} {\text{H}}_{ 5} {\text{OH}} + 1.5 {\text{CO}}_{ 2}$$

2 $${\text{C}}_{ 6} {\text{H}}_{ 12} {\text{O}}_{ 6} \to 2 {\text{C}}_{ 2} {\text{H}}_{ 5} {\text{OH}} + 2 {\text{CO}}_{ 2}$$

### Hydrocarbon biofuel production: acetic acid to hydrocarbon biofuel

The second section in the process starts with acetic acid fermentation broth and converts it into several intermediates, including ethyl acetate, ethanol and ethylene, before finally ending with a hydrocarbon end product. These process steps are outlined in Fig. 4.

The reactive distillation unit operation plays a key role in the overall biorefinery. This area combines two pieces of equipment, a reactor and a distillation column. The reaction of interest is the creation of ethyl acetate from acetic acid and ethanol, which occurs in the reaction section of the column. This reaction takes place at slightly acidic conditions by the addition of carbon dioxide, which forms calcium carbonate as the calcium acetate is protonated to form acetic acid. The bottom, or stripping section, leads to a reboiler. By constantly purging the reboiler, calcium carbonate that precipitates is removed from the system. In the top section, an azeotropic mixture of ethyl acetate, water, and ethanol is boiled off and condensed, thus driving the reaction forward [31]. The azeotrope is broken by pressure swing distillation, first by entering a high pressure distillation column. The bottoms of the high pressure column contain a purified ethyl acetate stream, which is sent on to hydrogenation, while the tops contain an azeotrope with greater proportions of water and ethanol. The ethanol is recovered for recycling into the process.

Ethyl acetate is hydrotreated, yielding two moles of ethanol for every one of the ester. This reaction occurs in the vapor phase. About half of the ethanol produced in the hydrogenation is recycled to the reactive distillation step, while the remaining half goes forward to biofuel production.

To achieve a hydrocarbon from ethanol, the olefin ethylene is formed via the catalyzed dehydration of ethanol. Next, compressed ethylene is dimerized to 1-butene. In a real process, the next step would create a spectrum of hydrocarbon molecules, but for the sake of modeling a 12-carbon jet fuel surrogate molecule is synthesized that contains a carbon–carbon double bond. Finally, the unsaturated hydrocarbon is hydrotreated to a saturated end product. The hydrocarbon biofuel is water, sulfur, and nitrogen free. The overall yield of hydrocarbon jet biofuel from hybrid poplar biomass is 330 L t^−1^.

### Hydrocarbon biofuel production: modeling the process

Modeling begins by examining the basic series of unit operations necessary for the biorefinery process. An Aspen flowsheet is then developed following the process described above, and known process parameters such as conversion percentages and losses are added. All major reactions are modeled with an RStoic block, and the literature values are used for operating temperature and pressure, fractional conversion data, and heat of reaction where applicable. Yields and conversion factors are conservative estimates in collaboration with our industry partners.

An overall reaction summary of the process is shown in Table 9 along with theoretical and practical yields. Due to incomplete conversion, selectivities lower than 100 %, and recovery losses, practical yields are lower than theoretical. During pretreatment, a sulfuric acid charge of 0.011 g g^−1^ of dry biomass is used and the operation occurs at 200 °C. 75 wt% of xylan is converted to xylose during pretreatment. Enzymatic hydrolysis occurs at 50 °C, and 91 wt% of cellulose is converted to glucose. Enzymes are assumed to be added at 20 mg g^−1^ cellulose [18]. During fermentation, 94 wt% of glucose [31] and 92 wt% of xylose are converted to acetic acid. The hydrolysis and fermentation conversion numbers are based on personal communication with industry partners (Tim Eggeman, personal communication, 2014), and are in line with proprietary data from commercial partners.

**Table 9:** Hydrocarbon biofuel production reaction network

Step	Chemistry	Cumulative theoretical yield	Cumulative practical yield
Pretreatment and hydrolysis	${\text{Wood}}\;{ + }\;{\text{H}}_{ 2} {\text{O}} \to {\text{Sugars}}\;{ + }\;{\text{Lignin}}$	722 kg Sugars	640 kg Sugars
Fermentation	${\text{C}}_{ 6} {\text{H}}_{ 12} {\text{O}}_{ 6} \to 3 {\text{CH}}_{ 3} {\text{COOH}}$	722 kg Acetic acid	580 kg Acetic acid
Hydrotreat (1)	${\text{CH}}_{ 3} {\text{COOH}} + 2 {\text{H}}_{ 2} \to {\text{CH}}_{ 3} {\text{CH}}_{ 2} {\text{OH}} + {\text{H}}_{ 2} {\text{O}}$	554 kg Ethanol	450 kg Ethanol
Alcohol dehydration	${\text{CH}}_{ 3} {\text{CH}}_{ 2} {\text{OH}} \to {\text{C}}_{ 2} {\text{H}}_{ 4} {\text{ + H}}_{ 2} {\text{O}}$	337 kg Ethylene	260 kg Ethylene
Oligomerization	$n {\text{C}}_{ 2} {\text{H}}_{ 4} \to {\text{CH}}_{ 3} {\text{CH}}_{ 2} - \left( {{\text{CH}}_{ 2} {\text{CH}}_{ 2} } \right)_{n - 2} - {\text{CH = CH}}_{ 2}$	337 kg Distillate	250 kg Distillate
Hydrotreat (2)	${\text{CH}}_{ 3} {\text{CH}}_{ 2} - \left( {{\text{CH}}_{ 2} {\text{CH}}_{ 2} } \right)_{n - 2} - {\text{CH = CH}}_{ 2} {\text{ + H}}_{ 2} \to {\text{CH}}_{ 3} {\text{CH}}_{ 2} - \left( {{\text{CH}}_{ 2} {\text{CH}}_{ 2} } \right)_{n - 2} - {\text{CH}}_{ 2} {\text{CH}}_{ 3}$	341 kg Polymer jet (450 L t^−1^)	250 kg Polymer jet (330 L t^−1^)

The major process steps are shown with the relevant chemical equations. Theoretical and practical yields shown use a basis of one bone dry tonne of poplar. Yields are based on the literature and conservative assumptions in collaboration with our industry partners

In reactive distillation, the reaction proceeds with an excess amount of ethanol and it is assumed that all acetic acid is consumed in ethyl acetate production. Ethanol yield from ethyl acetate hydrotreatment is 99.5 wt% for the entire unit operation. On a single pass basis, conversions of 90 % or higher are achieved [32]. The hydrotreating reaction runs in an adiabatic reactor and is slightly exothermic. The steps of reactive distillation, azeotrope separation, and ethyl acetate hydrotreating are combined in Table 9 to show the yield of ethanol from acetic acid.

For ethylene production from ethanol, an adiabatic fixed bed reactor system is assumed to be used. The process is industrially well established [33] with several commercial processes that have known conversion and selectivity rates to ethylene [34]. A scrubber column is used to remove water, followed by compression in a multi-stage compressor to 3.450 MPa with condensed water leaving the system. An adsorbent is used to remove final traces of water from the ethylene stream, modeled as a Sep block in ASPEN.

The two oligomerization reactors, to produce butene from ethylene and a 12-carbon molecule from butene, are cooled. The second of these two reactors is modeled in a loop with a distillation column (RadFrac). The column’s tops are recycled for further oligomerization, while the bottoms cut is in the jet fuel range and goes to a hydrotreatment reactor. A value of −124 kJ per mol is used for the exothermic reaction during hydrotreating to make a saturated end product [35].

It should be noted that the hydrocarbon end product, or polymer jet fuel, produced in modeling is a jet fuel surrogate. As a petroleum-derived product, jet fuel itself is a mixture of a large number of different hydrocarbons. Because the focus of this research is on the techno-economics of producing a biomass-based jet fuel, not specifically the modeled jet fuel product, one molecule is chosen as the jet fuel end product. n-Dodecane, C_12_H_26_, acts as a first approximation surrogate for kerosene-type jet fuel, and has an acceptable carbon number of 12 [36, 37]. The carbon number is important for calculating the degree of oligomerization required. Dodecane also has similar density, viscosity, thermal conductivity, and heat capacity to kerosene-based jet fuel [36]. It is, therefore, chosen as the end product to be modeled.

The simulation has a range of characteristics that make modeling challenging. For example, the model starts with a polar, water-based stream and ends with a non-polar hydrocarbon stream. In the intermediate, a ternary azeotrope of water, ethanol, and ethyl acetate is broken with pressure swing distillation. Thermodynamic and transport property estimation methods play an essential role in determining the accuracy of a simulation model. To select the proper Aspen property method, general guidelines set out by Aspen are followed and applied to each unit operation separately. The model’s base method is NRTL, with some unit operations operating under other property packages based on the material running through the process and Aspen guidelines for choosing a property method.

### Hydrogen production and auxiliary systems

**Fig. 5:**
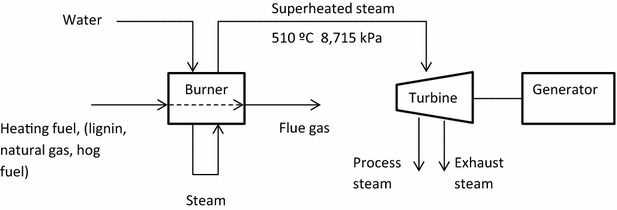
Generalized steam plant for biorefinery. Various scenarios are considered for the source of the heating medium, including lignin, natural gas, and hog fuel. In all cases, the minimum amount of steam is generated that is required to run the biorefinery

Hydrogen production comprises a special case in the biorefinery of interest, and links with process heat, steam, and electricity production. Various scenarios of hydrogen production are described. In all the scenarios, one central burner–boiler system provides heat and steam for the entire biorefinery and hydrogen production facility. Steam is generated at 8.715 MPa and 510 °C, and run through a turbine to produce lower temperature steam necessary for the process. In this way, electricity is generated for biorefinery consumption. A generalized diagram of the steam system is shown in Fig. 5.

An analysis of the heat duties in the model is performed so that process heat may be recycled where possible, to maintain high plant efficiency. Steam and electricity production are explicitly modeled and integrated into the biorefinery.

The hydrogen requirement in this biorefinery is a unique characteristic and provides many opportunities for optimization of the overall plant. Hydrogen production can be achieved by any number of sources [38–40]; however, three hydrogen sources are initially considered for investigation: electrolysis of water, lignin gasification, and natural gas steam reforming.

### Hydrogen production and auxiliary systems: electrolysis of water

The first source of hydrogen uses electricity, preferably from excess wind and hydro power, to produce the minimum required amount of H_2_ through electrolysis. Based on a 75 % efficient electrolysis system, the amount of electricity required for hydrogen production is 52.5 kWh per kg H_2_ [40, 41].

While electrolysis has appealing aspects, especially with regard to carbon emissions, a preliminary calculation of the cost of hydrogen production shows that the overall process is economically prohibitive. Even if electricity could be purchased at 0.03 USD kWh^−1^, producing the required amount of hydrogen would be the equivalent of 0.20 USD L^−1^ of hydrocarbon biofuel in electricity expenses alone. Due to this high cost and the availability of other, more economically viable options, no further investigation of hydrogen production using electrolysis is performed.

### Hydrogen production and auxiliary systems: lignin gasification

Lignin is often a derivative product of ethanol production that is burned to produce process heat and steam [18, 29, 42]. This fraction of the biomass entering the biorefinery may be gasified to produce hydrogen, thereby maintaining a higher percentage of the biomass in the final fuel product. However, if lignin is gasified, there is then a lack of heating medium to produce process heat and steam.

**Fig. 6:**
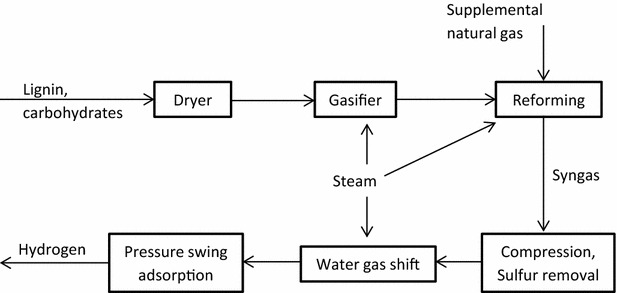
Generalized flow diagram of lignin gasification process. Gasifying lignin does not provide enough hydrogen for the biorefinery process, so a minimum amount of natural gas is reformed to make up the balance. Pressure swing adsorption is used to purify the hydrogen product, the same technology that is used in other hydrogen production scenarios considered in the present analysis

Although studies have investigated gasification of whole biomass for hydrogen production [39, 43], there exists little information on lignin gasification. Using literature sources for guidance, a process model is created in Aspen to generate data on lignin gasification for hydrogen production via syngas composed of carbon monoxide, carbon dioxide, and hydrogen. As a starting point, the compositional analysis of lignin and tar, a gasification intermediate, is found as shown in Table 10 [44]. The lignin feed stream also contains some carbohydrates as a result of the separations processes used in the biorefinery. An additional 4 % of the dry lignin weight is carbohydrate, primarily cellulose with slight amounts of hemicellulose. Overall lignin feed to the gasification facility is assumed to contain 50 % moisture. A generalized block flow diagram of the lignin gasification process is shown in Fig. 6.

**Table 10:** Compositional analysis of lignin, tar, and ash assumed to enter lignin gasification process [33, 40]

Component	% C	% H	% O	% Ash	Empirical formula
Lignin	53.7	6.6	36.5	3.1	${\text{CH}}_{ 1. 47} {\text{O}}_{ 0. 51}$
Tar	66.2	7.45	26.4	–	${\text{CH}}_{ 1. 35} {\text{O}}_{ 0.3}$
Ash	–	–	–	100	–

Beginning the gasification process, the lignin feedstock is first dried from 50 to 10 % moisture and preheated. The feedstock is then gasified with steam to produce 10 % char (solid carbon), 10 % tar, 2.9 % ash and 77.1 % gases. Steam is used as the gasification medium instead of oxygen or air, since hydrogen is our only desired product. Gasification occurs at just above atmospheric pressure and 870 °C. The amount of ammonia produced is negligible. Next, both char and ash are removed via a cyclone and combusted to recover heat for drying and preheating the lignin. The remaining gaseous product undergoes tar reforming to create a crude synthesis gas, or syngas. Syngas is a combination of hydrogen, carbon monoxide, and carbon dioxide, with a small amount of methane.

The lignin and small amount of carbohydrates do not provide enough hydrogen required by the poplar to jet process. To account for the difference, a natural gas steam reformer is added to create additional syngas, which is mixed with the biomass-based syngas stream. On a weight basis, the amount of natural gas added is equal to about 9 % of the total incoming lignin and carbohydrate stream.

The mixed syngas stream is cooled and compressed to 3.40 MPa and 50 °C for further processing. A liquid phase redox system that utilizes a chelated iron solution (LO-CAT) removes hydrogen sulfide, which will poison water gas shift (WGS) reaction catalysts. The redox system uses a readily available and regenerative catalyst and no toxic chemicals [45]. The sulfur content of lignin biomass is projected to much higher than conventional biomass due to the use of sulfur-based chemicals in the biorefinery pretreatment process; therefore, LO-CAT alone cannot remove enough hydrogen sulfide to avoid water gas shift catalyst poisoning. Even at hydrogen sulfide concentrations as low as 0.1 mg kg^−1^ the catalysts might start to deactivate [39]. Thus, a zinc oxide bed is used following the LO-CAT unit to further remove hydrogen sulfide from the gas stream.

High temperature (350 °C) and low temperature (200 °C) WGS reactors then convert most of the remaining carbon monoxide and steam into hydrogen gas and carbon dioxide. Catalysts are required for both high and low temperature reactors, consisting normally of iron oxide and chromium oxide, and copper oxide and zinc oxide, respectively [39].

The resulting gases with low carbon monoxide content (roughly 70 % hydrogen purity) are then cooled and demisted before entering a pressure swing adsorption (PSA) unit. It is essential to remove the entrained liquids (water and condensed hydrocarbons) because they will permanently damage the PSA adsorbent, which is a mixture of activated carbon and zeolite. It is also important that the gases are at a relatively lower temperature before entering the PSA column, since the equilibrium capacity of a molecular sieve decreases with increasing temperature and fewer impurities are adsorbed and removed. Purifying streams with original hydrogen purity less than 70 % may decrease the resulting product purity and overall hydrogen recovery [39]. The purified hydrogen stream leaving the PSA unit contains 85 % of the incoming hydrogen; there is an 85 % recovery rate of the hydrogen. The molar purity of hydrogen gas produced is 99.9 % or higher. The offgas, or reject stream of the PSA containing a majority of carbon dioxide along with other gases, is combusted in for heat energy recovery.

In total on a weight basis, about 91 % of the feed for hydrogen production is from the lignin and carbohydrate stream. The balance, about 9 %, is from natural gas. This compares to about 75 % of the syngas being derived from the biomass, while about 25 % is derived from natural gas. The overall yield hydrogen yield from dry feed is about 0.075 kg kg^−1^, which compares to 0.083 kg kg^−1^ for the gasification and processing of whole hybrid poplar chips [39].

### Hydrogen production and auxiliary systems: lignin gasification modeling

Lignin gasification includes nonpolar or mildly polar mixtures of hydrocarbon and light gases like hydrogen sulfide, carbon dioxide and hydrogen. Redlich–Kwong–Soave cubic equation of state with Boston–Mathias alpha function is the property method used in calculating the thermodynamic and transport properties such as fugacity coefficient, viscosity, and enthalpy. It was chosen because it is recommended for gas processing applications [46]. Since there is no thermodynamic data available specifically for lignin, tar, and ash in the Aspen Plus database, they were defined as nonconventional components using compositional data shown in Table 10. Pseudo-streams containing nitrogen and sulfur are also included in the simulation to simulate the formation of sulfur- and nitrogen-based compounds, especially hydrogen sulfide and ammonia.

An RStoic block is used to model drying the lignin feed. A calculator block is used to control the fractional conversion, so that the overall moisture of lignin goes down from 50 to 10 %. The evaporated water vapor is then separated from the lignin stream using a FLASH2 block and it is separated for heat recovery. The heat required for drying is recovered from char combustion. In addition to lignin drying, a calculator block is used to control the mass flow rate of elemental sulfur in the conventional solid stream such that it amounts to 3 % of the total wet lignin weight to simulate sulfur compounds that would be bound to the lignin stream. Another calculator block is also used to control the nitrogen mass flow rate in N_2_, so that it is always 0.01 times the wet lignin mass. These numbers are derived from the sulfur and nitrogen in the lignin stream from the pretreatment component of the Aspen simulation.

In gasification, an RYield block is first used to stimulate the decomposition of lignin into carbon, hydrogen, oxygen, tar, ash and char. Ash is removed, while char is combusted, due to its buildup in the recirculating heating medium. The carbon, hydrogen, tar and oxygen, along with steam (240 kPa, 130 °C), are sent to an RGibbs block and gasified. RGibbs provides a good approximation for the final product composition through Gibbs free energy minimization when the exact reaction mechanisms and kinetics are not well known. However, RGibbs is not capable of predicting the formation of solid char (solid carbon) and tar (black liquefied mixtures of phenols, polycyclic aromatic hydrocarbons, heterocyclic compounds, and other hydrocarbons); hence RYield is used first to convert 10 % of the overall lignin weight into water and 10 % of the dry lignin into tar, another 10 % into char with specified yield and composition, 2.9 % into ash and the remaining dry lignin mass into carbon, hydrogen gas and oxygen gas (Eq. 3). Sensitivity blocks are used to find the steam and reaction temperature that result in optimal hydrogen yields. Equation 4 shows the overall conversion of lignin and steam into hydrogen and carbon monoxide. The tar reformer is set up similarly to the gasifier: an RYield first decomposes all the tar into carbon, hydrogen and oxygen (Eq. 5), after which they are sent to RGibbs and reformed with steam (240 kPa, 130 °C). A stoichiometric amount of water is used to reform tar into carbon monoxide and hydrogen, shown by the overall reaction in Eq. 6.

3 $${\text{CH}}_{1.47} {\text{O}}_{0.51 } ({\text{Lignin}}) \to {\text{C}} + 0.735{\text{H}}_{2} + 0.255{\text{O}}_{2}$$

4 $${\text{CH}}_{ 1. 47} {\text{O}}_{0.51 } \left( {\text{Lignin}} \right) + 0.49{\text{H}}_{ 2} {\text{O}} \to 1.225{\text{H}}_{2} + {\text{CO }}$$

5 $${\text{CH}}_{ 1. 35} {\text{O}}_{0.3} ({\text{Tar}}) \to {\text{C}} + 0.675{\text{H}}_{ 2} + 0.15{\text{O}}_{ 2}$$

6 $${\text{CH}}_{ 1. 35} {\text{O}}_{0.3} ({\text{Tar}}) + 0.7{\text{H}}_{ 2} {\text{O}} \to 1.375{\text{H}}_{2} + {\text{CO }}$$

In sulfur removal modeling, the gas is desulfurized into two RStoic blocks, simulating the LO-CAT reactor and zinc oxide bed. The reactions involved in reoxidizing the LO-CAT solution using air (in which hydrogen sulfide is oxidized to elemental sulfur and water) are simplified in Eq. 7. The remaining hydrogen sulfide is removed via zinc oxide bed (Eq. 8) modeled using an RStoic block. It is assumed that any remaining sulfur is present only in negligible quantities.

7 $${\text{H}}_{ 2} {\text{S}} + \frac{1}{2}{\text{O}}_{2} \to {\text{S}} + {\text{H}}_{ 2} {\text{O }}$$

8 $${\text{ZnO}} + {\text{H}}_{ 2} {\text{S}} \to {\text{ZnS}} + {\text{H}}_{ 2} {\text{O }}$$

Both high and low temperature water gas shift reactors with 350 and 200 °C reactor temperatures, respectively, are modeled as REquil with zero temperature approach [39]. Approach temperature is defined as the difference between the measured outlet temperature and the temperature that would yield the measured conversion of a component at equilibrium (in our case, carbon monoxide) [39]. It is assumed the reaction is at equilibrium and, thus, temperature approach is zero. Since the water gas shift reaction itself is slightly exothermic, a lower reactor temperature tends to result in better yield but slower reaction and vice versa. This explains the use of high and low temperature reactors instead of just one WGS reactor. Hydrogen gas separation and purification are performed in a PSA unit modeled as a Sep2 block, with split fractions specified to achieve expected product yield and selectivity [39, 47].

### Hydrogen production and auxiliary systems: natural gas steam reforming

The most common method of producing hydrogen commercially, natural gas steam reforming [41], also referred to as steam methane reforming (SMR), uses two primary reactions to form hydrogen from hydrocarbons. Methane, being the primary component of natural gas, and steam are reformed to produce syngas. To increase hydrogen yields from natural gas, the syngas then goes through low and high temperature WGS reactions. The resulting gasses then go through a pressure swing adsorption system to obtain a nearly pure hydrogen product.

SMR is a widely used, widely studied, advanced and mature production process [38, 41, 47, 48]. The bulk of the data for the SMR process simulation is derived from an Idaho National Lab report [38]. The overall SMR process has a cold gas efficiency of 76.6 % (higher heating value basis), and produces a hydrogen yield of 0.35 kg kg^−1^of natural gas feed. This hydrogen production method is commercially well developed. It is explored in detail, and has been combined with a carbon capture system in other studies [38, 48], although that is out of the scope of this study.

In the SMR model, the reformer is modeled as an RGibbs block, and the high and low temperature WGS reactions are modeled as REquil blocks using methods similar to those described for lignin gasification. A PSA unit is used as the final hydrogen separations and purification operation, and is used as described previously.

### Hydrogen production and auxiliary systems: scenario selection of hydrogen production

Oxygen must be removed from the process stream to make a hydrocarbon end product from biomass and intermediates that contain oxygen. Overall chemical reduction in the process is provided by hydrogen gas and the oxygen is ultimately removed in the form of water. How the hydrogen is obtained can play a major role in the overall techno-economics.

In this paper, we consider two scenarios. In the first case, hydrogen is produced primarily by lignin gasification. Natural gas is reformed as a supplement to lignin gasification to produce just enough hydrogen for the two hydrotreating reactions to make jet fuel. Similarly, only enough natural gas is used in the burner and boiler system to provide steam and heat for the wood to jet process and the gasification process. In the second case, SMR is used to provide all the necessary hydrogen. All the available lignin is sent to the burner, where it provides a majority of the heating value. Additional heating value comes from methane containing biogas from the waste water treatment system, and natural gas or hog fuel for any balance of remaining heat requirements.

### Economic modeling

#### Economic modeling: capital expenses

The capital cost is estimated using a well-known method of factored estimation, and may include uncertainties of as much as ±30 % [18, 42]. The method starts with costs of major pieces of equipment based on the literature or software estimation. Using the flow data generated during process simulation, the cost of major pieces of equipment is scaled based on Eq. 9. The flow components, Flow_a_ and Flow_b_, are simply mass, heat, or work data generated from simulation. The scaling exponent, n, is found in the literature and is different for various types of equipment, but usually is in the range of 0.5–0.8. For example, the scaling exponents were 0.6 for the boiler, 0.6 for the pretreatment reactor, 0.7 for the pre-fermentation solids separator, and 1.0 for the fermentors [18]. An additional file shows the scaling exponent for the reactor, column, or primary piece of equipment for each major unit operation (see Additional file 1).

9 $${\text{Cost}}_{\text{a}} = {\text{Cost}}_{\text{b}} \times \left( {\frac{{{\text{Flow}}_{\text{a}} }}{{{\text{Flow}}_{\text{b}} }}} \right)^{\text{n}}$$

The scaled cost is then multiplied by an installation factor, and updated to 2014 USD using the Chemical Engineering Plant Cost Index. Besides the conversion plant that produces jet fuel from biomass, waste water treatment, a cooling tower system, a burner/boiler/generator system, and hydrogen production are included in the final capital expense.

The installed equipment comprises the bare module cost, which is then multiplied by a factor of 1.68 to include additional costs—contingency, contractor fee, site development, auxiliary buildings, off-sites, and utilities [49]. The final estimated capital expense is grassroots, which refers to a completely new facility although it does not include the cost of land.

#### Economic modeling: operating expenses

Operating expense estimation is done as follows: A list of inputs and outputs of the biorefinery model is compiled. This includes raw materials entering the biorefinery, and waste and byproducts exiting the facility. Costs are associated with each item, based on the literature sources or publicly available industry quotes. Some costs, such as enzymes, are not narrowly defined but may be estimated based on literature [50]. Fixed costs, which include labor, maintenance, overhead, administration, and various other support activity costs, comprise around 20 % of the cash cost to produce the biofuel and are estimated based on Turton et al. [49]. Labor costs are estimated by the number of employees and salary, while other fixed costs are estimated by factors of labor or capital. Any excess electricity is to be sold to a nearby grid at 0.06 USD kWh^−1^, although the excess amount is small. An electricity price of 0.06 USD is within the range of historical wholesale prices in the U.S. [51] with a 0.023 USD kWh^−1^ production tax credit [52]. Based on simulation results, a cost per hour, year, or volume of hydrocarbon fuel may be estimated.

#### Economic modeling: discounted cash flow rate of return analysis

One way to measure and compare the profitability of investments, a discounted cash flow rate of return, or internal rate of return analysis takes into account an entire project timespan. In summary, the analysis manipulates the jet fuel selling price to find the break-even point at which the project net present value is zero. This calculation is performed by iteration with a specified discount rate, and the final price is the minimum jet fuel selling price on a volume basis. The minimum selling price is the minimum price that the fuel must be sold for in order to break even under the assumed discount rate. Selling the product for a higher price will increase the internal rate of return, while selling for a lower price will reduce the rate of return.

Key parameters of the analysis are shown in Table 11. The two discount rates are chosen for two different purposes. First, a 7 % discount rate is chosen as a baseline for social opportunity cost of capital, based on appropriate discounting from a benefit–cost analysis point of view [53]. A higher, 15 % discount rate is used for comparison and reflects a discount rate that would be more attractive to investors. The minimum selling price uses a pre-tax position (0 % tax rate). A pre-tax calculation was chosen because the tax requirement for a given biorefinery is unclear. In addition, with cellulosic biofuels being an emerging industry, there is the potential for substantial government subsidy and tax breaks.

**Table 11:** Discounted cash flow analysis parameters

Parameter	Value
Discount rates (nominal, compounded yearly)	7, 15 %
Project lifetime (plant operation)	20 years
Construction time	3 years
Equity share	100 %
Tax rate	0 %
Working capital (% of fixed capital investment)	10 %, returned at project completion

## Additional file

**Additional file 1.** Temperature and pressure of major unit operations from Aspen modeling, and the capital scaling factor.

## Abbreviations

APR: annual percentage rate
Btu: British thermal unit
Kg: kilogram
L: liter
PSA: pressure swing adsorption
SMR: steam methane reforming
USD: United States Dollar
WGS: water gas shift
